# Adenoma mimicking intraductal papillary neoplasm of the bile duct arising in an intrahepatic biliary duplication cyst

**DOI:** 10.1093/bjrcr/uaae012

**Published:** 2024-04-10

**Authors:** Yuichi Tsuji, Shimada Kotaro, Hiroyoshi Isoda, Takamichi Ishii, Yasuhide Takeuchi, Yuji Nakamoto

**Affiliations:** Department of Diagnostic Imaging and Nuclear Medicine, Graduate School of Medicine, Kyoto University, Kyoto 606-8507, Japan; Department of Diagnostic Imaging and Nuclear Medicine, Kyoto University Hospital, Kyoto 606-8507, Japan; Department of Diagnostic Imaging and Nuclear Medicine, Kyoto University Hospital, Kyoto 606-8507, Japan; Department of Hepato-Biliary-Pancreatic Surgery and Transplantation, Kyoto University Hospital, Kyoto 606-8507, Japan; Department of Diagnostic Pathology, Kyoto University Hospital, Kyoto 606-8507, Japan; Department of Diagnostic Imaging and Nuclear Medicine, Kyoto University Hospital, Kyoto 606-8507, Japan

## Abstract

We report a case of a cystic liver tumour in a 47-year-old man with Peutz-Jeghers syndrome (PJS) who had undergone sclerotherapy at another hospital for a cyst in hepatic segment IV (S4) 7 years earlier. Based on the preoperative imaging findings, the patient was diagnosed with an intraductal papillary neoplasm of the bile duct. Percutaneous transhepatic portal vein embolization was performed to increase the residual liver volume, followed by resection of the three right hepatic lobes and the caudate lobe, biliary reconstruction, and portal vein reconstruction. Pathological examination revealed an adenoma arising in an intrahepatic biliary duplication cyst. Retrospectively, the preoperative diagnosis was difficult, but it aligned with previous reports of biliary duplication cysts due to its continuity with the bile duct. Additionally, intrahepatic biliary duplication cysts with tumour lesions or cases in which 18F-fluorodeoxyglucose positron emission tomography was performed have not been previously reported. Therefore, preoperatively listing this disease as a differential diagnosis was difficult. PJS and chronic inflammation associated with cyst sclerotherapy may have contributed to tumour development in the intrahepatic biliary duplication cyst.

## Clinical presentation

A 47-year-old man with Peutz-Jeghers syndrome (PJS) underwent sclerotherapy (ethanol and minomycin) at another hospital for a cyst in hepatic segment IV (S4) 7 years earlier. Additionally, the patient had a history of surgical resection of intestinal polyps in 2021 and multiple other endoscopic procedures for small-bowel polyps. In January 2022, a papillary protuberance appeared on the wall of the hepatic cyst and a relatively rapid growth trend suggested malignancy. The patient was referred to our hospital for surgical resection.

## Investigations

**Figure 1.**
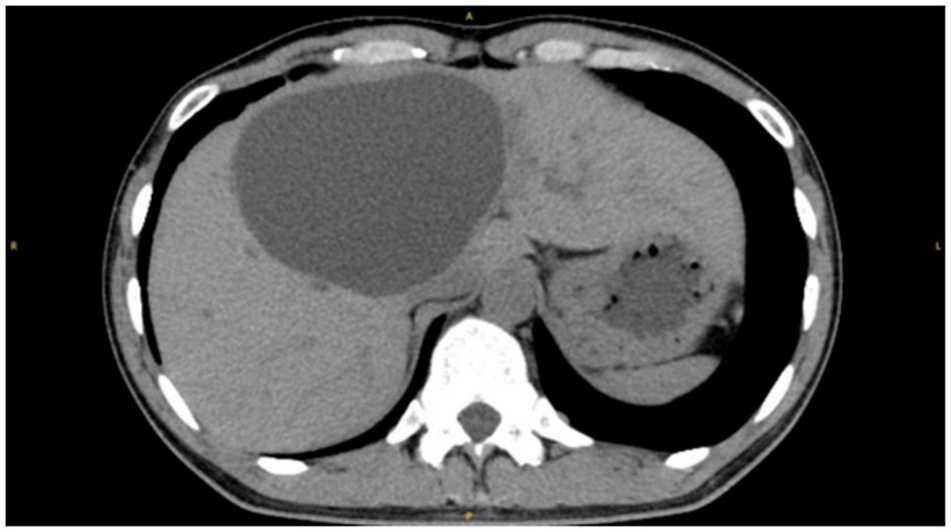
Non-enhanced computed tomography of the upper abdomen shows a large cyst in hepatic S4.

**Figure 2.**
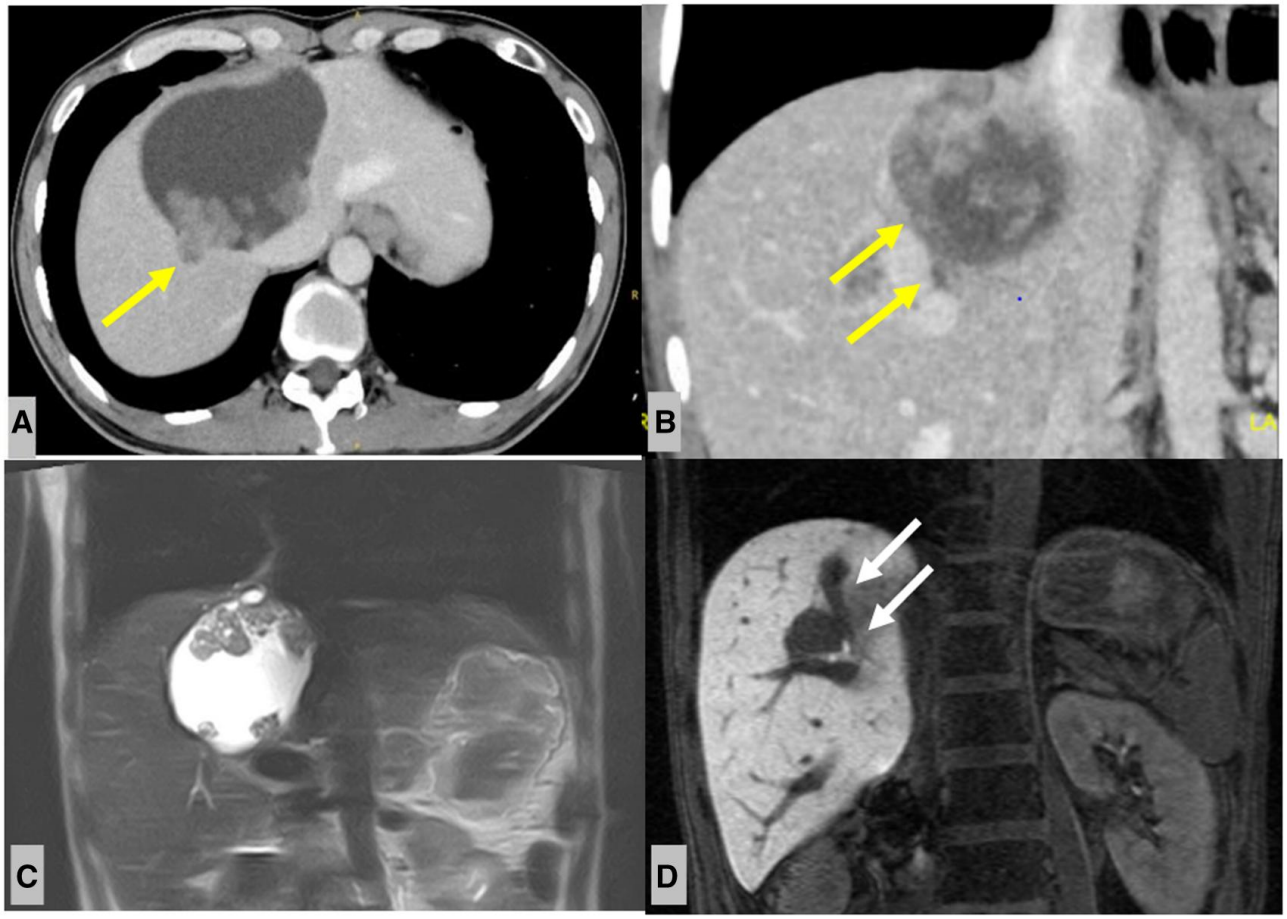
(A, B) Contrast-enhanced CT of the upper abdomen (one year after non-enhanced CT). Axial and coronal sections show several solid components in the hepatic cyst, and extension into the B7 bile duct (yellow arrow). (C) Coronal T2-weighted magnetic resonance image shows several solid components within the hepatic cyst. (D) Coronal hepato-biliary phase shows infiltration into intrahepatic bile duct B7, as suspected by CT imaging (white arrow). Abbreviation: CT = computed tomography.

Non-contrast CT revealed a large hepatic cyst in hepatic S4 (Figure 1). Contrast-enhanced CT and MRI 1 year later showed a large cystic mass with numerous papilla-like solid portions located in hepatic S4 (Figure 2). Part of the mass extended into a tubular structure that appeared to be the bile duct and was thought to be a bile duct tumour plug. No dilated intrahepatic bile ducts were observed on CT or MRI.

**Figure 3.**
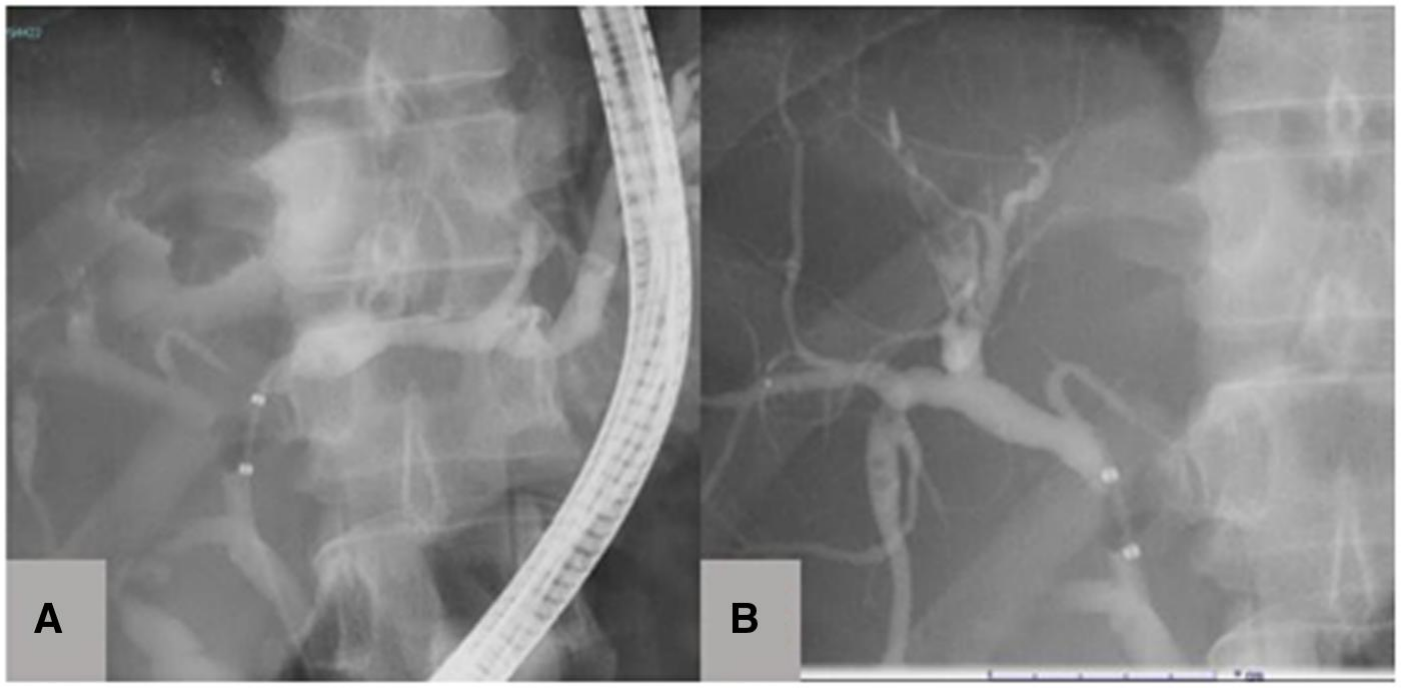
Cholangiography shows (A) contrast medium influx into the hepatic cyst (white arrow) and (B) contrast defects suggestive of a bile duct tumor plug (yellow arrow).

**Figure 4.**
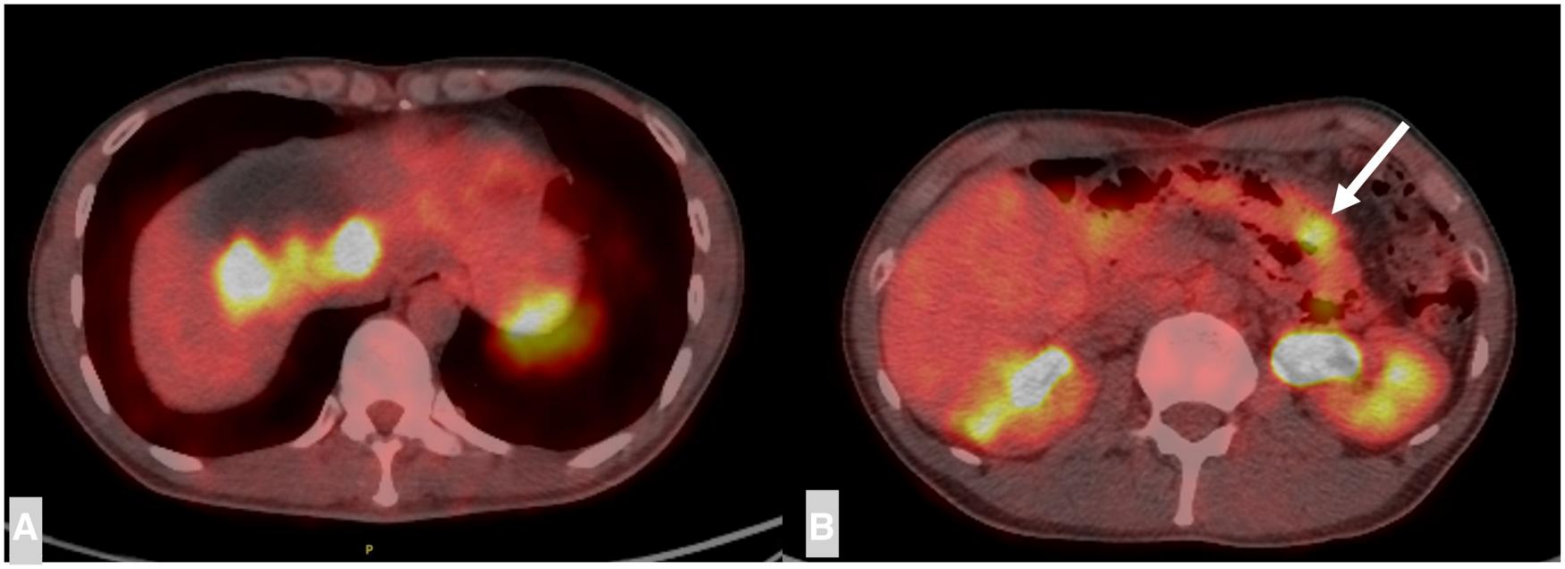
FDG-PET/CT of the axial image shows (A) FDG accumulation consistent with the solid components within the hepatic cyst and (B) FDG accumulation are also observed in a part of the small intestine (white arrow). Abbreviations: FDG = 18F-fluorodeoxyglucose; PET = positron emission tomography; CT = computed tomography

Cholangiography revealed an inflow of contrast medium into the tumour area from the left hepatic duct, suggesting communications (Figure 3A). In addition, the bile duct in the right anterior segment was stretched and narrowed by the tumour, and a defect was observed in the dilated B7 bile duct (Figure 3B), which corresponded with the bile duct tumour plug on CT. PET revealed moderate to intense uptake of 18F-fluorodeoxyglucose (FDG), corresponding to the solid portion of the cystic liver mass (Figure 4A), with a maximum standardized uptake value of 7.9. PET-CT also showed nodular accumulation in the small intestine, which was suspected to be accumulation in hamartomatous polyps (Figure 4B). No metastases were detected in other sites.

Based on these findings, the patient was diagnosed with an intraductal papillary neoplasm of the bile duct (IPNB).

## Treatment

Percutaneous transhepatic portal vein embolization was performed to increase the residual liver volume, followed by resection of the three right hepatic lobes and the caudate lobe, biliary reconstruction, and portal vein reconstruction. Some small intestinal tumours were also resected simultaneously. Extensive adhesions because of the previous surgeries were noted in the upper and lower abdominal cavity, and lesion continuity with the duodenum was unclear.

## Diagnosis

**Figure 5.**
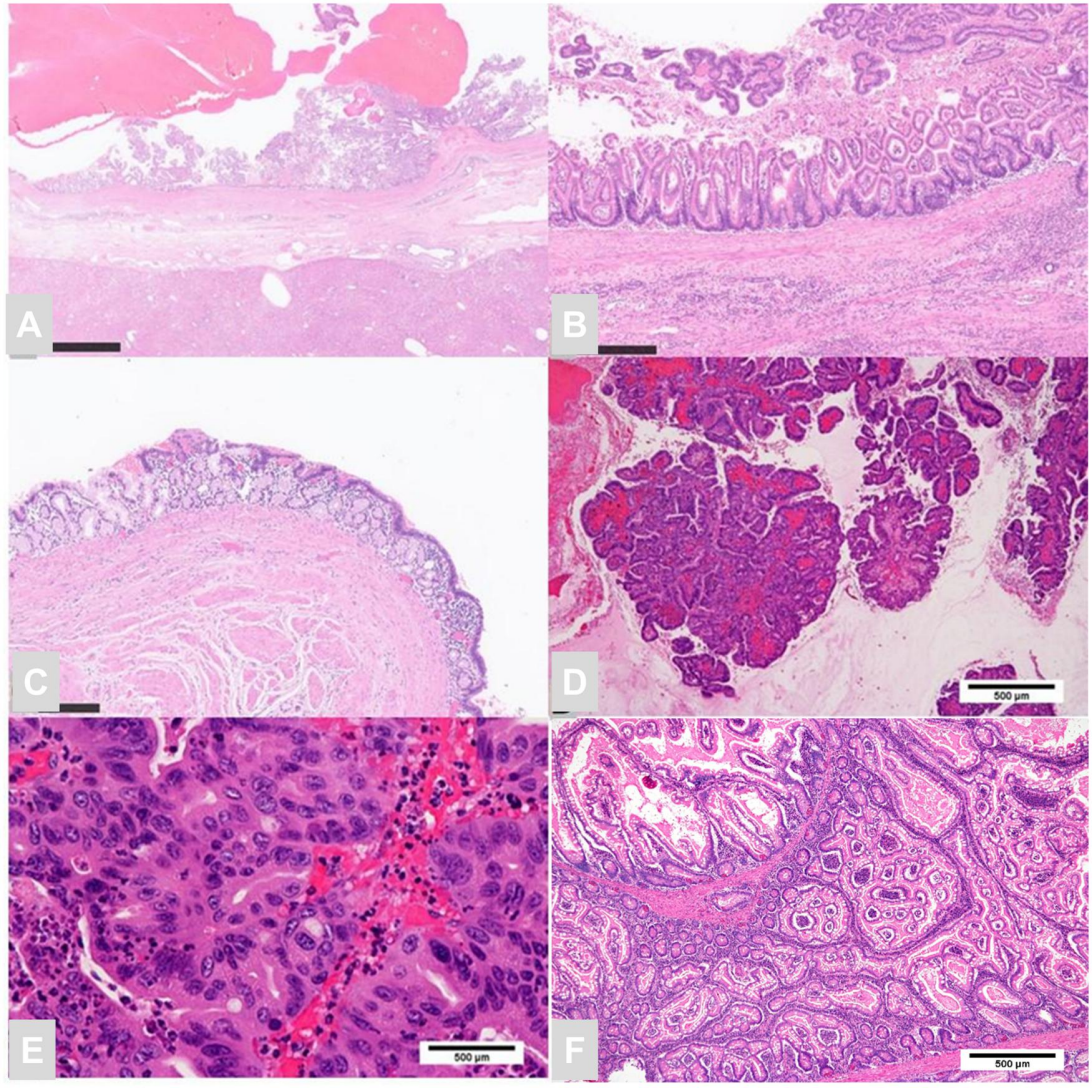
Histological sections of the hepatic cyst. (A) The cyst is surrounded by thick smooth muscle. (B, C) The intracystic mucosa resembles that of the glandular gastric pylorus and small intestine. (D, E) Gastric-type adenoma with high-grade dysplasia. The resected small intestinal tumour shows papillary proliferation of hyperplastic-like epithelium, consistent with the pathological findings of a hamartomatous polyp (F).

Gross examination revealed a yellowish-white solid papillary lesion within the cyst. Histologically, the cyst was covered with a highly differentiated epithelium resembling that of the stomach and duodenum and was surrounded by a thick smooth-muscle layer (Figure 5A-C). The tumour within the cyst was an adenoma with gastric-type phenotype, and few regions showed high-grade cellular and structural atypia (Figure 5D and E). Based on these findings, a diagnosis of high-grade adenoma arising from a biliary duplication cyst was made. The small intestinal tumours were diagnosed as hamartomatous polyps (Figure 5F).

## Outcome, follow-up

No evidence of recurrence was observed at 8 months postoperatively.

## Discussion

Duplications of the digestive tract are rare conditions that can occur anywhere from the oral cavity to the anus. These duplications are surrounded by smooth muscle layers and are diagnosed by the presence of digestive tract mucosa. They no longer require continuity with the conventional digestive tract.^1^

Biliary duplication cysts are extremely rare, accounting for less than 1% of all gastrointestinal duplication cysts.^1^ In the English literature, only five cases have been reported, with Lee et al reporting the first case in a 13-year-old male in 1957.^2^ Previously reported cases have predominantly involved young individuals aged 8-18 years,^2–5^ although a case has been reported in a 51-year-old man. Symptoms of biliary duplication cysts include pain, diarrhoea, jaundice, loss of appetite, and fatigue, attributed to bile duct obstruction and inflammation caused by digestive fluid originating from the digestive tract mucosa.^1^

The ideal treatment for duplication cysts is complete surgical excision, given the rarity of malignant transformation. Reported cases have described choledochal cyst with inflammation in young patients or gallbladder duplication. In one case that was discovered in a 51-year-old man, excision was performed without a preoperative diagnosis.

In the present case, a malignant liver tumour was suspected because of the presence of a papillary enhancing lesion inside the cystic mass, its relatively rapid growth, and intense FDG uptake in the solid part. The tumour was found to communicate with the bile ducts, with tumour extension into these ducts, similar to imaging findings in IPNB. While consistent with previous reports in terms of biliary continuity, its asymptomatic nature prompted further consideration of the possibility of a tumour.

The cause of the tumour inside the biliary duplication cyst was unknown; however, the patient had a history of PJS. PJS is an autosomal-dominant hereditary disease characterized by hamartomatous polyps of the gastrointestinal tract and mucocutaneous melanin deposits. PJS is commonly caused by germline mutations in the tumour suppressor genes LKB1/STK11 on chromosome 19. Previous studies have reported a significantly increased risk of gastrointestinal and extraintestinal malignancies in patients with PJS. Although the malignant transformation mechanism is not fully understood, the hamartoma-adenoma-carcinoma sequence has been proposed as a mechanism for tumour malignancy.^6^ This case also appears to have followed this sequence. To the best of our knowledge, biliary duplication cysts in patients with PJS have not been reported, suggesting this may be an incidental finding. However, we hypothesized that PJS may have contributed to tumour development. It is also possible that chronic inflammation associated with cyst sclerosis therapy may have contributed to tumour development. To our knowledge, this is the first report of a tumour arising from a biliary duplication cyst with PJS. PJS is associated with a significantly increased risk of gastrointestinal malignancies, and previous cyst sclerosis therapy-related chronic inflammation may be associated with the development of intracystic tumours.

## Learning points

- Intrahepatic biliary duplication cysts are extremely rare. And this is the first report of a tumour arising within an intrahepatic biliary duplication cyst.
- Peutz-Jeghers syndrome and chronic inflammation associated with cyst sclerotherapy may contribute to tumour development in intrahepatic biliary duplication cysts.

## References

[1] randle RW , Qasem SA, Shen P. Biliary duplication cyst. Am Surg. 2015;81(7):E291-E293. 26140880

[2] Lee CM. Jr Duplication of the cystic and common hepatic ducts, lined with gastric mucosa. N Engl J Med. 1957;256(20):927-931. 13451969 10.1056/NEJM195705162562003

[3] Kim J , Jarboe MD, Arnold MA, DiPietro MA, Bloom DA, Teitelbaum DH. Biliary duplication cyst with heterotopic gastric mucosa resulting in obstruction of the biliary system: a case report. J Pediatr Surg. 2012;47(6):E5-E8. 10.1016/j.jpedsurg.2012.01.066 22703824

[4] Akers DR , Favara BE, Franciosi RA, Nelson JM. Duplication of the alimentary tract: report of three unusual cases associated with bile and pancreatic ducts. Surgery. 1972;71(6):817-823. 5030497

[5] Grumbach K , Baker DH, Weigert J, Altman RP. Biliary tract duplication cyst with gastric heterotopia. Pediatr Radiol. 1988;18(4):357-359. 3387162 10.1007/BF02389016

[6] Bosman FT. The hamartoma-adenoma-carcinoma sequence. J Pathol. 1999;188(1):1-2. 10398131 10.1002/(SICI)1096-9896(199905)188:1<1::AID-PATH327>3.0.CO;2-J

